# Increased crystalloid fluid requirements during zone 3 Resuscitative Endovascular Balloon Occlusion of the Aorta (REBOA) versus Abdominal Aortic and Junctional Tourniquet (AAJT) after class II hemorrhage in swine

**DOI:** 10.1007/s00068-020-01592-x

**Published:** 2021-01-30

**Authors:** Andreas Brännström, Albin Dahlquist, Jenny Gustavsson, Ulf P. Arborelius, Mattias Günther

**Affiliations:** 1 Department of Clinical Science and Education, Södersjukhuset, Karolinska Institutet, Sjukhusbacken 10, S1, SE-118 83 Stockholm, Sweden; 2 Department of Neuroscience, Karolinska Institutet, Stockholm, Sweden

**Keywords:** Non-compressible hemorrhage, Resuscitation, Aortic tourniquet, REBOA, Prehospital care

## Abstract

**Purpose:**

Pelvic and lower junctional hemorrhage result in a significant amount of trauma related deaths in military and rural civilian environments. The Abdominal Aortic and Junctional Tourniquet (AAJT) and infra-renal (zone 3) Resuscitative Endovascular Balloon Occlusion of the Aorta (REBOA) are two options for resuscitation of patients with life threatening blood loss from and distal to the pelvis. Evidence suggest differences in the hemodynamic response between AAJT and zone 3 REBOA, but fluid management during resuscitation with the devices has not been fully elucidated. We compared crystalloid fluid requirements (Ringer’s acetate) between these devices to maintain a carotid mean arterial pressure (MAP) > 60 mmHg.

**Methods:**

60 kg anesthetized and mechanically ventilated male pigs were subjected to a mean 1030 (range 900–1246) mL (25% of estimated total blood volume, class II) haemorrhage. AAJT (*n* = 6) or zone 3 REBOA (*n* = 6) were then applied for 240 min. Crystalloid fluids were administered to maintain carotid MAP. The animals were monitored for 30 min after reperfusion.

**Results:**

Cumulative resuscitative fluid requirements increased 7.2 times (mean difference 2079 mL; 95% CI 627–3530 mL) in zone 3 REBOA (mean 2412; range 800–4871 mL) compared to AAJT (mean 333; range 0–1000 mL) to maintain target carotid MAP. Release of the AAJT required vasopressor support with norepinephrine infusion for a mean 9.6 min (0.1 µg/kg/min), while REBOA release required no vasopressor support.

**Conclusion:**

Zone 3 REBOA required 7.2 times more crystalloids to maintain the targeted MAP. The AAJT may therefore be considered in a situation of hemorrhagic shock to limit the need for crystalloid infusions, although removal of the AAJT caused more severe hemodynamic and metabolic effects which required vasopressor support.

## Introduction

Trauma is a major global health issue contributing to about 10% of overall mortality and an annual worldwide death of more than 5.8 million people [1, 2]. Hemorrhage is still the leading cause of potentially preventable death among trauma victims [2, 3]. Non-compressible hemorrhage from the abdomen, pelvis, junctional regions and proximal lower extremities constitutes a particular challenge since exsanguination can occur rapidly and conventional interventions are ineffective [4]. In the military setting, 19% of haemorrhage-related deaths 2001–2011 occurred from the junctional regions only, emphasizing an injury pattern of growing importance in armed conflicts [5–7].

Resuscitative endovascular balloon occlusion of the aorta (REBOA) and the Abdominal Aortic and Junctional Tourniquet (AAJT) are devices with the potential to control hemorrhage from the lower body including the pelvis by closing off arterial inflow [8–10]. Occlusion of the aorta may also benefit resuscitation by providing proximal hemodynamic support and reduce the requirement of resuscitative fluids. The AAJT is designed for external compression of the abdominal aorta resulting in infra-renal (zone 3) occlusion while the endovascular REBOA balloon can be positioned at any level within the aorta. Zone 3 REBOA and the AAJT are equally effective in achieving hemostasis [11]. For patients with traumatic hemorrhage, current guidelines in the US recommend a zone 3 location of the REBOA balloon for isolated injuries including and distal to the pelvis [12]. Both devices constitute potential prehospital and battlefield interventions to decrease mortality from non-compressible torso hemorrhage. In conflict environments, the AAJT may be the only realistic option to stabilize and evacuate a casualty with massive non-compressible hemorrhage below the aortic bifurcation [13]. One major benefit of the non-invasive AAJT is the ease of use compared to the REBOA technique which requires percutaneous or surgical femoral arterial access. However, the REBOA balloon may be a more suitable option in higher levels of care with the possibility to maintain hemostasis during surgery and adaptable reperfusion under vigorous hemodynamic monitoring. We have shown that a transition from the AAJT to zone 3 REBOA may be safely performed with hemodynamic support, in a report where we suggested hemodynamic differences between the interventions [14]. We have also shown that reduction of the perfused vascular volume by the AAJT reduced crystalloid fluid requirements to maintain vital circulation compared to fluid resuscitation only [8]. Although causing less severe ischemia and being better tolerated after reperfusion, zone 3 REBOA has limited resuscitative hemodynamic effects compared to a zone 1 location [15].

A recent animal study described improved blood pressure support when resuscitating with whole blood and fresh frozen plasma compared to crystalloids when comparing the AAJT and REBOA in a pooled group. The AAJT and REBOA animals did not differ in hemodynamics, metabolic responses or survival [16]. In contrast, our experience suggests that the AAJT and zone 3 REBOA lead to different hemodynamic situations, and that REBOA requires increased fluid resuscitation to maintain blood pressure.

A large volume of crystalloids is an independent factor for increased morbidity and mortality in trauma patients. Fluid resuscitation of patients with impending or manifest hemorrhagic shock should replace sufficient blood volume to maintain cardiac output and end-organ perfusion while preserving blood coagulation and oxygenation. Crystalloids address the first of these parameters while having a negative impact on coagulation and oxygen transportation [17, 18]. No comparison has been reported on crystalloid fluid management during zone 3 REBOA and the AAJT. We, therefore, compared crystalloid fluid requirements to maintain carotid MAP > 60 mmHg during a 4 h prolonged application of the AAJT and zone 3 REBOA after a class II (25%) hemorrhage.

## Materials and methods

**Figure 1:**
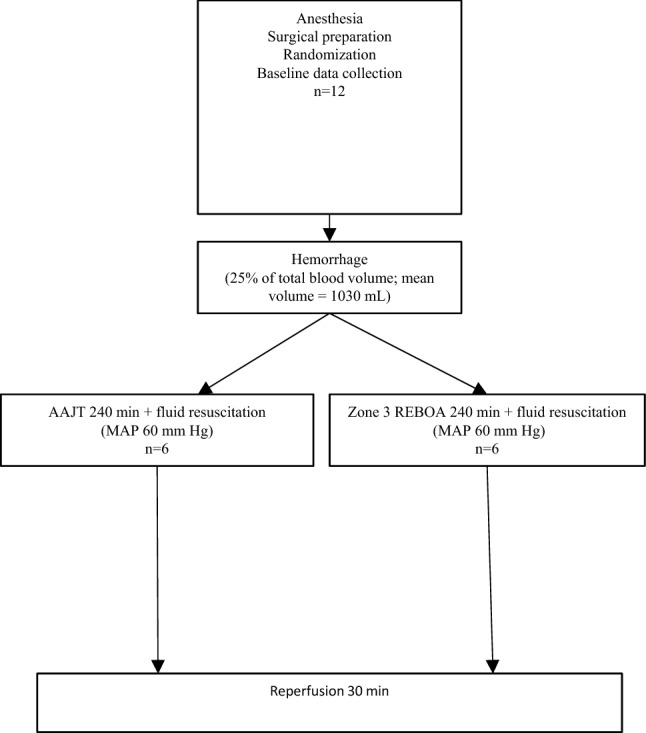
Experimental design

This study was approved and conducted in accordance with the Swedish regional ethics approval board for animal research (S3-15), and included four phases: animal preparation, hemorrhage, intervention (AAJT or zone 3 REBOA) and reperfusion (Fig. 1).

### Surgical preparation and instrumentation

Castrated, crossbred male pigs (54–65 kg) were pre-medicated with 150 mg tiletamine/zolazepam (Zoletil 100 Vet) and 6 mg medetomidine (Domitor) and placed supine on a standard operating table. 100% O_2_ was administered via a nose cone for three minutes before anesthesia induction through the right auricular vein with pentobarbital 6 mg/kg, atropine 0.02 mg/kg and 2.5 µg/kg fentanyl. Animals were orally intubated with an 8 mm endotracheal tube and mechanical ventilation (Hamilton C2 ventilator, Hamilton medical, Geneva Switzerland) was adjusted to PaCO_2_ 4.7–5.5 kPa. Initial ventilator settings were: inspiratory oxygen fraction 21%; peak inspiratory pressure 16 cm H_2_O and peak expiratory end pressure 4 cm H_2_O. Anesthesia was maintained by continuous infusion of ketamine 25 mg/kg/h (Ketaminol Vet 100 mg/ml), midazolam 0, 0485 mg/kg/h (Midazolam Hameln 1 mg/ml) and fentanyl 3.5 µg/kg/h (Fentanyl B. Braun 50 µg/ml). A fluid bolus of 500 ml Ringer’s acetate was given after induction of anesthesia to adjust baseline fluid-balance discrepancies and then continuously throughout the experiments with 3 ml/kg/h to replace insensible losses.

A 7.5 F Swan-Ganz pulmonary artery catheter (Edwards Lifescience) was introduced in the surgically exposed right external jugular vein and used for core temperature, cardiac output (CO) and mixed venous oxygen saturation (SvO_2_). The left carotid artery was surgically exposed and used for hemorrhage, hemodynamic monitoring and collection of blood samples. A suprapubic urine catheter and 12 EKG electrodes were placed to monitor heart rate, the occurrence of arrhythmias and to verify euthanasia. The AAJT was pre-positioned and left in place unbuckled. In the REBOA animals, a 7F introducer (REBOA Medical, Båstad, Norway) was placed via the femoral artery using Seldinger-technique.

### Induction of hemorrhage shock

A 25% of estimated total blood volume hemorrhage, modified from an earlier study with hemorrhage in two phases under hemodynamic monitoring, was utilized to create a reproducible state of class II hemorrhage [19]. TBV was estimated by 67 ml/kg. Initially 50% of calculated blood loss was drawn for 7 min by a peristaltic pump (Masterflex L/S, Cole Parmer) and 450 ml of shed blood was stored in citrated bags at 38 °C. After a 10 min stabilization of MAP, as a response to splenic autotransfusion, hemorrhage was continued at half the rate until target volume was achieved.

### Interventions

Animals were assigned to groups AAJT application (AAJT; *n* = 6) or zone 3 REBOA (REBOA; *n* = 6). After completion of hemorrhage the AAJT was inflated (300 mmHg) or a 15 mm balloon (REBOA Medical, Båstad Norway) was positioned in the infra-renal aorta guided by anatomical measurement on the skin from the site of vascular access to aortic bifurcation before insertion and inflated with 8 ml NaCl. We have verified anatomical measurement as a robust method of estimating the level of the balloon in our earlier experimental series [14]. A zone 3 location of the balloon was verified in all animals post-mortem via laparotomy. T_0_ was defined as when the AAJT or REBOA balloon was fully inflated. Complete data collection started at T_0_. Total occlusion of aortic blood flow was verified by loss of a pulse wave and no blood flow in a percutaneous arterial catheter placed distally to the devices, non-measurable noninvasive blood pressure on the hind leg, non-measurable heart rate (HR) and pulse-oximetry from the tail probe.

### Intravenous fluid resuscitation

In accordance with current Tactical Combat Casualty Care battlefield resuscitation guidelines [20] for situations with no access to whole blood, component therapy or colloids, Ringer´s acetate was administered in 250 ml bolus infusions as needed, following AAJT or REBOA inflation, if MAP < 60 mmHg. At 240 min the animals were transfused with 450 ml autologous blood from stored citrated bags until MAP approximated baseline. The AAJT or the REBOA balloon was then promptly deflated. Additional rapid crystalloid infusion of 2000 mL Ringer´s acetate was continued throughout the reperfusion phase in all animals. Norepinephrine (0.1 µg/kg/min) was infused if MAP < 60 mmHg. After 30 min, animals were euthanized by 40 ml pentobarbital sodium (Alfatal Vet, 100 mg/ml).

### Data-collection and statistical analyses

Hemodynamic performance and fluid administration were continuously registered and arterial blood gases and hemoglobin were collected. Arterial pressure was measured between 0–200 mm Hg and monitored as maximum (systolic), mean (automatically calculated) and minimum (diastolic). The following calculations were used for hemodynamic parameters: Stroke volume (SV) = CO/HR, SVR = 80 × (MAP−CVP)/CO [21]. Primary outcome was cumulative crystalloid fluid requirements to maintain MAP > 60 mmHg. Secondary outcomes were hemodynamic and metabolic (pH, base excess, lactate, hematocrit and core body temperature) parameters. A power calculation for a continuous outcome superiority trial required 6 animals per group to have an 80% chance of detecting a 2500 ml difference in the primary outcome (*α* = 0.05). Standard deviations were calculated from pilot studies. For five animals in the AAJT group, a secondary analysis of previously published data was performed [8] and one animal was added according to the power calculation.

All statistical analyses were made by GraphPad Prism version 7.03 for Windows (GraphPad Software). *p* < 0.05 was considered significant. All data are expressed as mean ± SD. Two-way ANOVAs with multiple comparisons tests were used for iv fluids, hemorrhage volume, hemodynamic- and metabolic parameters. Student’s unpaired t-test was used for norepinephrine.

## Results

**Figure 2:**
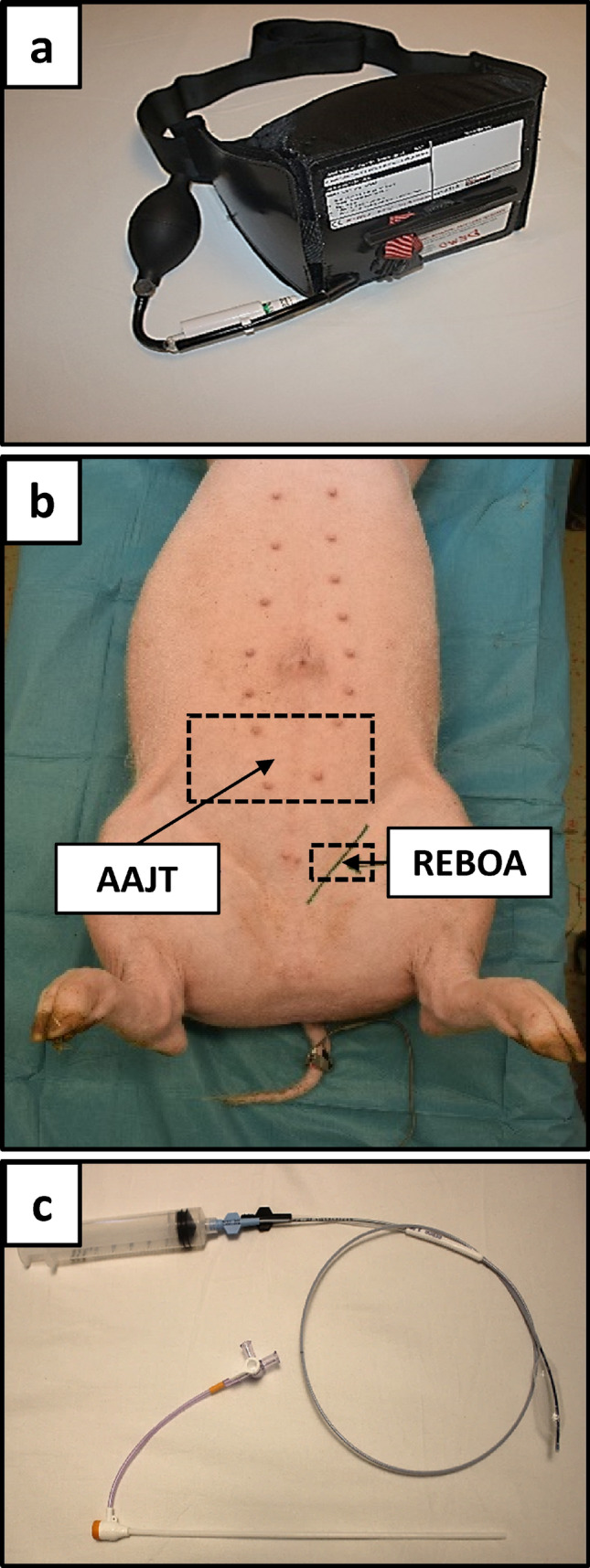
Abdominal view of a 60 kg laboratory animal **b** with anatomic landmarks for aortic occlusion devices; AAJT **a** and REBOA balloon catheter with 7.5 F introducer **c**

**Figure 3:**
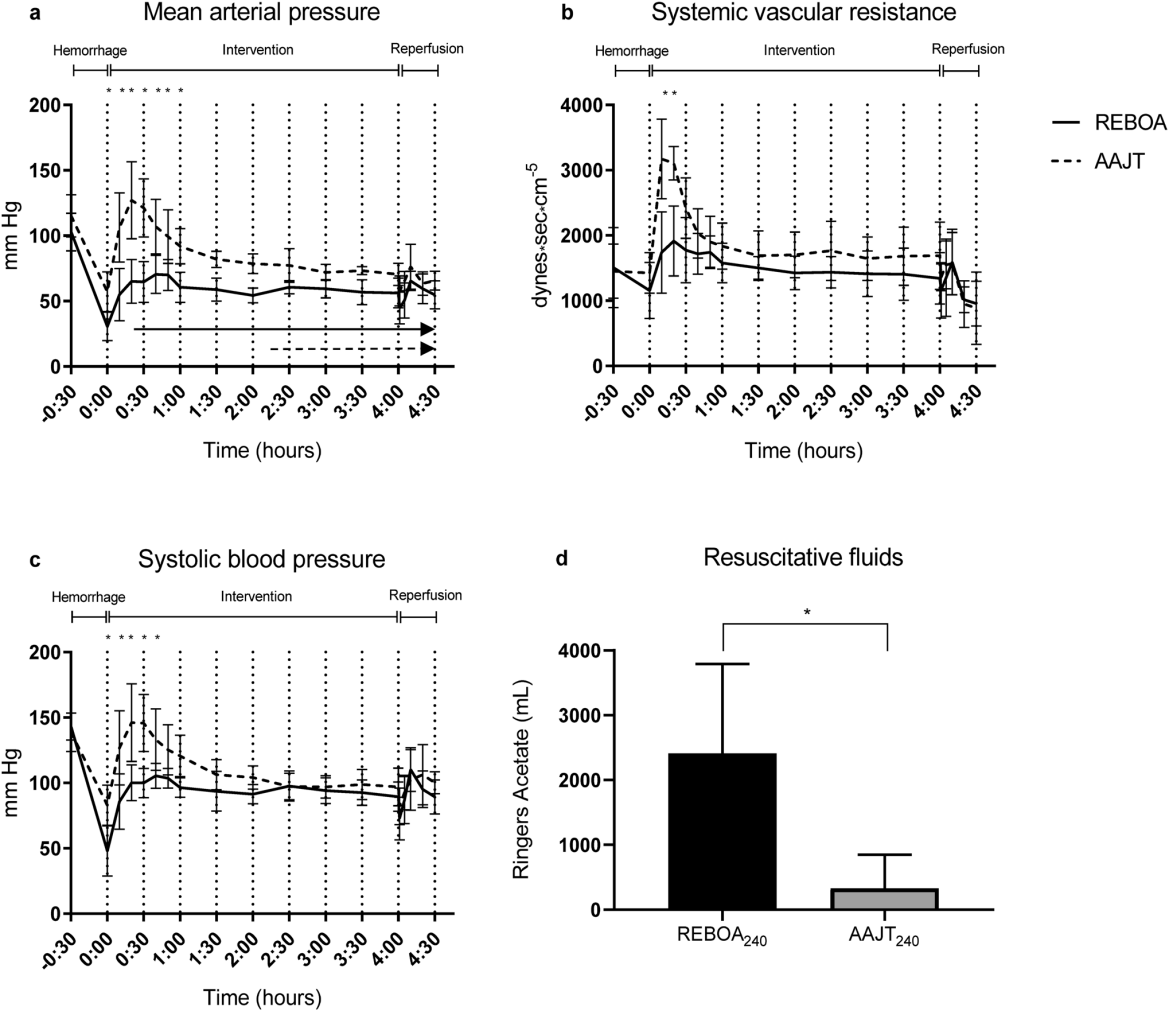
Hemodynamic changes during experiments **a–c** and cumulative crystalloid infusions between groups **d**. Arrows in **a** represents crystalloid infusion times. Data are presented as mean with standard deviation. A *p* < 0.05 was considered significant. *p < 0.05

Baseline characteristics displayed differences for MAP which was higher in the AAJT group and heart rate which was higher in the REBOA group (Table 1). Average blood loss was 980 ml (AAJT) and 1080 ml (REBOA) (Table 1). The hemorrhage protocol resulted in a MAP reduction of 50% (AAJT) and 70% (REBOA), respectively (Fig. 3). Interventions with AAJT or REBOA were performed according to the protocol with no complications or malfunctions of the devices (Fig. 2). The application time did not exceed one minute in both study groups.

**Table 1:** Baseline characteristics and hemodynamic parameters

	AAJT_240_	REBOA_240_	*p* value
Body weight (kg)	57.5	61.5	ns*
Hemorrhage (mL)	980 (168)	1080 (102)	0.25
Systolic blood pressure (mmHg)	139 (15)	143 (10)	>0.99
Mean arterial pressure (mmHg)	115 (16)	103 (14)	0.93
Heart rate (/min)	105 (22)	112 (34)	>0.99
Cardiac output (L/min)	6.4 (1.5)	6.15 (2.4)	>0.99
Stroke volume (mL)	61 (6)	56 (17)	0.998
Systemic vascular resistance (dynes*sec*cm^5^)	1453 (415)	1509 (614)	>0.99

Group means (SD), *not significant

**Figure 4:**
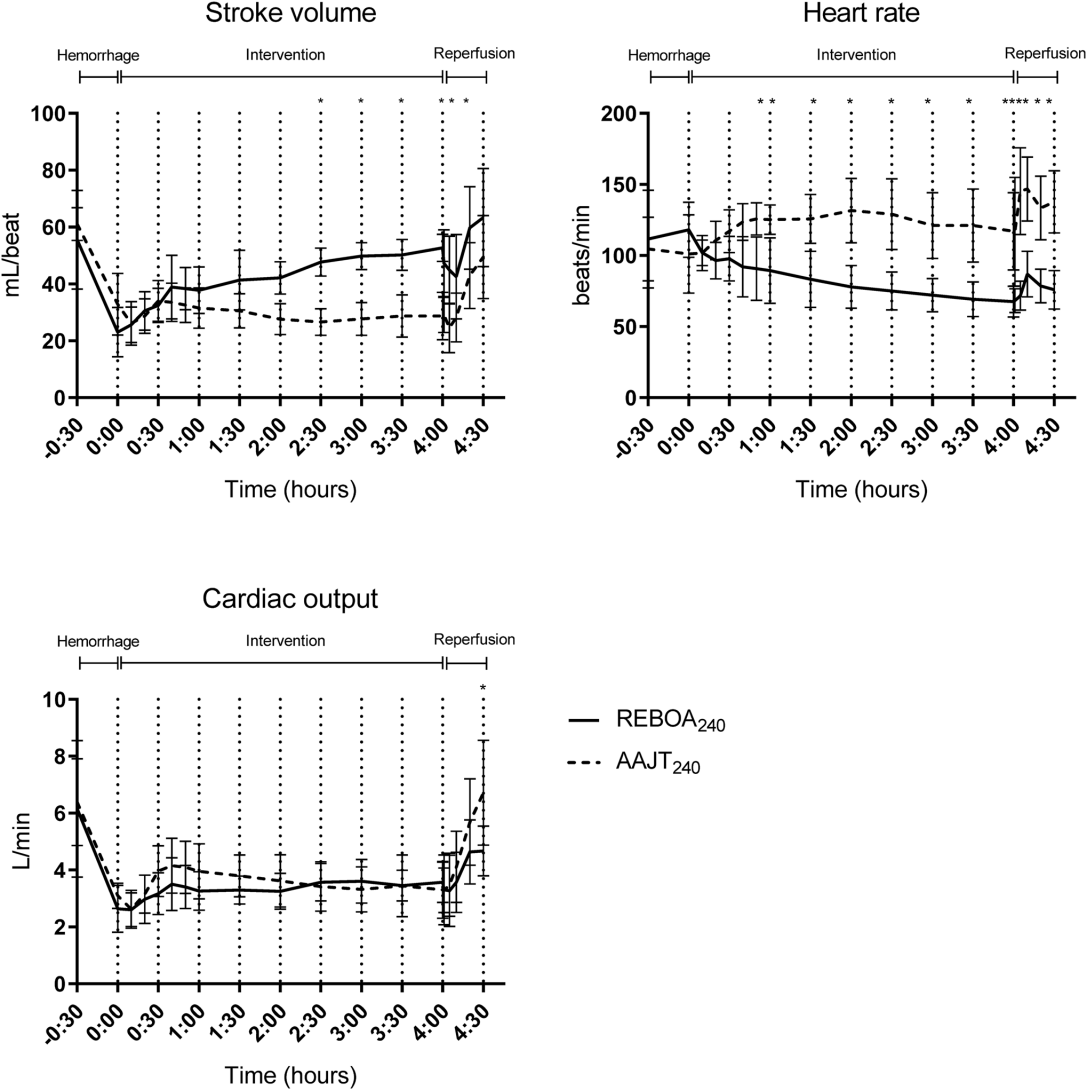
Cardiac performance between groups during experiments. Data are presented as mean with standard deviation. A *p* < 0.05 was considered significant. *p < 0.05

All animals survived the intervention and subsequent 30 min reperfusion phase. Both interventions caused a MAP increase of more than 100% (Fig. 3). The systolic blood pressure increased by 67% (AAJT) and 122% (REBOA), respectively (Fig. 3). Peak MAP and SBP occurred at T_20_ in the AAJT and T_40_ in the REBOA group (Fig. 3). After deflation of the AAJT and REBOA balloon, an expected decrease in MAP and SBP occurred within one minute. After nor-epinephrine infusion was started, MAP was kept > 60 and SBP > 90 mmHg. Average cumulative resuscitative fluids at 240 min were 2412 ml (range: 800–4800 mL) in the REBOA group and 333 ml (range: 0–1000 mL) in the AAJT group (Fig. 3). Mean difference in resuscitative fluids was 2079 mL (95% CI 627–3530 mL). Four animals resuscitated with the AAJT upheld a MAP > 60 mmHg without crystalloid fluids. Systemic vascular resistance (SVR) increased after both interventions but was significantly higher in the AAJT group between T_0_ and T_30_. After T_60_ SVR had little variation until reperfusion when it decreased, with no trend of stabilization (Fig. 3). Release of the AAJT required vasopressor support with norepinephrine infusion for a mean time of 9.6 min and a mean total of 55.2 µg (0.1 µg/kg/min), while REBOA animals required no vasopressor support. Cardiac output displayed no differences between interventions and showed a little increase after AAJT/REBOA balloon inflation (Fig. 4). After deflation of the AAJT/REBOA balloon cardiac output increased to supra-normal in the AAJT animals. Heart rates increased after AAJT application throughout the experiment with near 100% increase compared to REBOA (Fig. 4). After reperfusion heart rates increased further for five (AAJT) and ten (REBOA) minutes respectively. Stroke volume was significantly higher in the REBOA group during the intervention phase (Fig. 4). After deflation, SV increased simultaneously in both study groups after five (AAJT) and ten (REBOA) minutes. No signs of ventricular fibrillation or prolonged Q-T interval appeared on EKG during the experiments.

**Figure 5:**
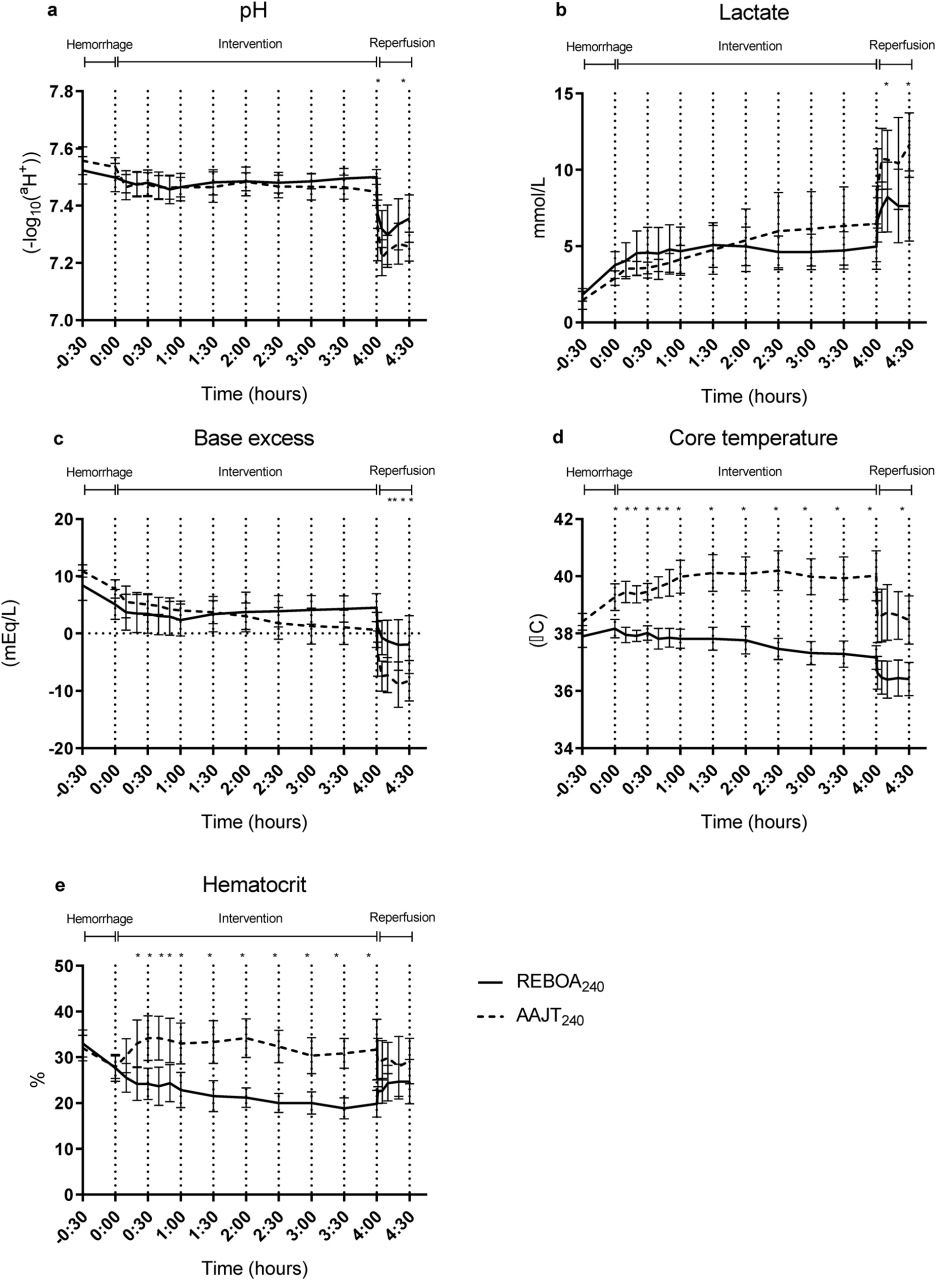
Multipanel figure of metabolic changes during experiments. Data are presented as mean with standard deviation. A *p* < 0.05 was considered significant. * = *p* < 0.05

Hematocrit increased in the AAJT group compared to REBOA. AAJT application caused an increase of hematocrit by 17% within the first 30 min, whereafter it decreased towards baseline (Fig. 5). REBOA application caused a decrease in hematocrit by 27% after hemorrhage, until autologous transfusion and reperfusion were performed. Lactate showed a slight and simultaneous increase in both groups after hemorrhage until T_90_ with a responding decrease in base excess (Fig. 5). Between T_150_ and end of experiment, lactate was significantly higher in the AAJT animals. Both interventions caused a sharp increase in lactate after deflation. Core body temperature increased in AAJT animals compared to REBOA animals which in contrast were hypothermic (Fig. 5). Deflation of the devices caused an immediate decrease in temperature in both groups with no trend towards normalization.

## Discussion

In this study we demonstrate differences in crystalloid fluid requirements between zone 3 REBOA and the AAJT to maintain a target MAP. It is possible that a zone 3 REBOA offers limited hemodynamic support without blood volume replacement in hemorrhagic shock, compared to AAJT, which may affect the applicability of the devices in different clinical situations.

Based on the effect on hemodilution and hypothermia which we observed when comparing AAJT with crystalloid fluid resuscitation only [8], we aimed to demonstrate a 2500 mL difference in crystalloid fluids between the devices. In remote battlefield or civilian prehospital environments this amount may represent a near exhaustion of available resuscitative fluids. Although the study did not reach 2500 mL assumed in the power analysis, the difference of 2079 mL suggests clinically important differences in crystalloid fluid management between the interventions. Importantly, only two animals in the AAJT group required fluid resuscitation in addition to the device application to maintain vital perfusion.

The differences in hemodynamics between the devices are likely explained by an interruption of splanchnic circulation by the AAJT with a subsequent increased systemic vascular resistance. Furthermore, compression of the vena cava and reduced cardiac preload likely explain**s** the elevated heart rates and reduced stroke volumes in the AAJT animals although both groups had similar cardiac output. While our findings contrast a study by Rall et al. where no significant hemodynamic differences were reported except for carotid MAP [11], Tibbits et al. compared hemodynamic effects of zone 1 to zone 3 REBOA and also reported limited hemodynamic support from zone 3 REBOA [15]. The AAJT increases the intra-abdominal pressure and therefore higher ventilator pressures may be expected during mechanical ventilation. The hemodynamic impact of increased intrathoracic pressures during positive pressure ventilation should be addressed in future studies.

A complete separation of blood flow between tissues proximal and distal to the AAJT likely caused more severe ischemic insults after reperfusion, with an accumulation of anaerobic metabolites causing increased metabolic acidosis after device removal. Vasopressor support after AAJT removal was not required after zone 3 REBOA, and suggested a more severe ischemic injury in the AAJT group. The increased amount of crystalloid fluids to maintain MAP above 60 mmHg in the REBOA animals likely explained the difference in hematocrit between groups. It is possible that REBOA animals where diluted while AAJT animals had extravascular plasma shift from the increase in blood pressure. In addition, the significantly lower core body temperature in the REBOA animals may have caused coagulopathy. Future studies addressing fluid resuscitation in conjunction with aortic occlusion devices should include analyses of coagulation. We found no significant differences in the metabolic parameters pH, lactate and base excess during the intervention which implied adequate resuscitation. Worth noting is the differing composition of Ringer’s lactate and Ringer´s acetate, the latter principally used in Scandinavia. The theoretical advantage of acetate as a buffer is the systemic metabolism compared to the liver-dependent lactate metabolism. Large volumes of Ringer´s lactate have been associated with increased lactate levels compared to acetate solutions while not affecting the pH [22], which is why it is likely that Ringer’s acetate did not cause any significant changes in lactate levels in the present study.

From a prehospital point of view, possible complications associated with REBOA must be emphasised. The tolerance of the device is limited by ischemia. In addition; arterial access, catheter insertion and balloon positioning risk vascular injury, unsuccessful resuscitation and vital organ ischemia [23]. Furthermore, the correct location of the balloon is crucial but may be hard to determine, and the possibility to monitor the physiology in austere environments may be challenging [24]. REBOA may therefore not be a feasible option in battlefield conditions, compared to the AAJT.

Current guidelines limit AAJT application to 60 min to avoid permanent ischemic injuries [25–27]. We applied a prolonged application time to investigate crystalloid fluid requirements for 240 min. The application time was chosen by the authors based on their experience and a possible scenario of delayed definite treatment after major bleeding in an emergency scenario. Robust preclinical hemodynamic data may guide fluid resuscitation in such an event. Prolonged application may occur in complex environments such as in battlefields which raises practical and ethical considerations. A successful prehospital resuscitation and evacuation of a patient with the AAJT may also warrant transition to zone 3 REBOA during surgical treatment which constitutes another rationale for investigating the fluid requirements beyond 60 min. Kheirabadi et al. demonstrated permanent ischemic injuries to the spinal cord after AAJT application exceeding 60 min. Spinal cord injuries have not been studied after prolonged REBOA [12, 25] why studies addressing safe time limits for zone 3 REBOA are needed. An important finding of the present study were increased pH and base excess and lower lactate after prolonged zone 3 REBOA, suggesting that the AAJT should be replaced with zone 3 REBOA when feasible, to avoid deteriorating ischemic effects.

The study has some limitations to be discussed. The swine model is frequently used in translational research on physiological response to hemorrhage and interventions, but the translation to humans should be done with caution. Another limitation is the method of haemorrhage by a controlled bleeding from the carotid artery. We did not perform measurements on coagulation, BE and pH on collected blood before auto-transfusion. The coagulation system may be activated differently in traumatic haemorrhage, which should be addressed in future studies. Citrate may affect the electrolyte- and metabolic balance in patients, especially after rapid and massive transfusions. However, impaired citrate metabolism in humans is mainly associated with liver disease, and administration of citrated blood was limited to one bag of 450 mL in animals with normal liver function. No hypocalcaemia was detected. Therefore, the influence of citrated blood transfusion likely had a negligible impact on the metabolic results. The study design allowed for comparisons of fluid resuscitation with a limited number of experimental animals, in accordance of the principle of reduction of experimental animals, from the principles of the 3Rs (Replacement, Reduction and Refinement), the framework for ethical animal research. However, as a consequence, the short observation time after reperfusion was also a major limitation. A longer intensive care phase would allow for assessments of organ function and general physiologic consequences including inflammation, coagulation and renal failure. The increased vasopressor need after removal of the AAJT is likely caused by redistribution of blood from the ischemic vasculature distal to the AAJT together with a reperfusion injury with ensuing inflammatory response and capillary leakage. It is unclear how and to what extent these mechanisms contribute, and an assessment would require a longer follow-up, which would also allow for investigations of postoperative fluid management. Future studies should therefore include a longer reperfusion phase, covering the first 24 h.

We did not assess central venous pressure (CVP). To correctly assess the central venous blood volume indicated by CVP, the intra-thoracic pressure from positive pressure ventilation should have been equal between groups. The AAJT applies pressure towards the diaphragm causing higher ventilator pressures to maintain adequate tidal volumes and gas exchange in the lungs, which is why CVP likely was related to ventilatory pressures. In this study, the animals were mechanically ventilated to maintain etCO_2_ within a normal range, and adjustments in ventilatory pressures were made accordingly. Future studies may investigate CVP in relation to the devices in spontaneous ventilation. Finally, the positive pressure mechanical ventilation was also a limitation since the effect on cardiac preload possibly differs between the interventions. Future studies may compare the impact of positive pressure ventilation and spontaneous ventilation when applying AAJT and zone 3 REBOA.

## Conclusion

Zone 3 REBOA required 7.2 times more crystalloids to maintain the targeted MAP. The AAJT may therefore be considered in a situation of hemorrhagic shock to limit the need for crystalloid infusions, although removal of the AAJT caused more severe hemodynamic and metabolic effects which required vasopressor support.
